# Supplementary material for: Evaluation of cell wall preparations for proteomics: a new procedure for purifying cell walls from Arabidopsis hypocotyls
Source: Plant Methods. 2006 May 27;2:10. doi: 10.1186/1746-4811-2-10 (PMC1524762; doi:10.1186/1746-4811-2-10)
Supplement: Additional data file 1 — Table 1 – Bioinformatic analysis of proteins extracted from cell walls of A. thaliana cell suspension cultures [8]. [file 1746-4811-2-10-S1.pdf]

Additional file 1: Bioinformatic analysis of proteins extracted from cell walls of *A. thaliana* cell suspension cultures [8].

Data are from [8]. Proteins were extracted from A. thaliana cell walls of cell suspension cultures as described in Figure 1. Two successive extractions were performed : step 1 using $CaCl_2$ and step 2 using urea. All proteins sequences were analyzed with three bioinformatic programs to predict their sub-cellular localization. Proteins for which predictions by different bioinformatic programs are in conflict are classified as "not clear".

step 1: $CaCl_2$ extract

Predicted subcellular localization	Gene (*A. thaliana*)	PSORT (a)	TargetP (b)	Predicted signal peptide (c)	Aramemnon (d)
**transmembrane domain**	At3g30810	Golgi (0.900), plasma membrane (0.790), chloroplast thylakoid membrane (0.762)	other (0.796)		yes
	At4g12420	plasma membrane (0.919)	secretory pathway (0.989)	1-20	GPI anchor
**outside**	At1g06870	outside (0.638)	secretory pathway (0.850)	1-21	
	At1g21670	outside (0.820)	secretory pathway (0.973)	1-21	
	At1g53070	outside (0.528)	secretory pathway (0.972)	1-17 or 1-23	
	At1g78830	outside (0.820)	secretory pathway (0.962)	1-22	
	At1g78850	outside (0.820)	secretory pathway (0.973)	1-22	
	At2g39700	outside (0.810)	secretory pathway (0.961)	1-20	
	At2g41800	outside (0.370)	secretory pathway (0.369)	1-21	
	At2g44450	outside (0.820)	secretory pathway (0.853)	1-22	
	At2g47050	outside (0.820)	secretory pathway (0.928)	1-20	
	At3g07320	outside (0.790)	secretory pathway (0.482)	1-19	
	At3g08030	outside (0.820)	secretory pathway (0.985)	1-21	
	At3g45960	outside (0.805)	secretory pathway (0.744)	1-20	
	At3g45970	endoplasmic reticulum (0.820)	secretory pathway (0.854)	1-20	
	At3g61820	outside (0.628)	secretory pathway (0.828)	1-26 or 1-25	
	At4g20830	outside (0.820)	secretory pathway (0.540)	1-30	
	At4g25900	outside (0.820)	secretory pathway (0.924)	1-20 or 1-24	
	At4g30270	outside (0.800)	secretory pathway (0.975)	1-21	
	At5g06860	outside (0.733)	secretory pathway (0.964)	1-21	
**intracellular**	At1g08450	endoplasmic reticulum (0.910) (C-terminal HDEL)	secretory pathway (0.995)	1-28	
	At1g13440	microbody (0.539)	other (0.610)		
	At1g30580	microbody (0.507)	other (0.579)		
	At2g39050	microbody (0.515)	other (0.910)		
	At2g43710	microbody (0.495)	chloroplast (0.977)		
	At3g02630	chloroplast stroma (0.593)	chloroplast (0.779)		
	At3g26060	mitochondry (0.679)	chloroplast (0.904)		
	At3g48380	mitochondry (0.596)	mitochondry (0.308)		
	At3g58480	nucleus (0.760)	other (0.802)		
	At4g17260	cytoplasm (0.450)	other (0.435)		
	At4g37870	nucleus (0.880)	other (0.849)		
	At5g08310	mitochondry (0.789)	mitochondry (0.946)		
	At5g20080	mitochondry (0.859)	mitochondry (0.855)		
	At5g46550	nucleus (0.940)	other (0.504)		
	At5g55990	microbody (0.569)	secretory pathway (0.580), other (0.576)		
**not clear**	At4g22410	cytoplasm (0.450)	secretory pathway (0.922)	1-26	
	At5g37990	endoplasmic reticulum (0.550)	secretory pathway (0.523), chloroplast (0.307)	1-15	

**step 2: urea extract**	**transmembrane domain**	At1g61500	plasma membrane (0.460)	secretory pathway (0.963)	1-24	yes
		At1g67880	plasma membrane (0.790)	other (0.955)		yes
		At3g25560	plasma membrane (0.460)	secretory pathway (0.742)	1-32	yes
		At3g57400	plasma membrane (0.830)	other (0.792)		yes
		At5g08390	plasma membrane (0.460)	secretory pathway (0.932)	1-22	yes
		At5g58640	plasma membrane (0.640)	secretory pathway (0.326)	1-17	yes
	**outside**	At1g71695	outside (0.820)	secretory pathway (0.829)	1-22 or 1-31	
		At2g16430	outside (0.820)	secretory pathway (0.963)	1-25	
		At2g44450	outside (0.820)	secretory pathway (0.853)	1-22	
		At3g08030	outside (0.820)	secretory pathway (0.985)	1-21	
		At3g45970	endoplasmic reticulum (0.820)	secretory pathway (0.854)	1-20	
		At3g52500	outside (0.820)	secretory pathway (0.649)	1-18	
		At4g08770	outside (0.633)	secretory pathway (0.955)	1-22	
		At4g24890	outside (0.820	secretory pathway (0.925)	1-20 or 1-26	
	**intracellular**	At1g24360	mitochondry (0.613)	chloroplast (0.545), mitochondry (0.206)		
		At1g30580	microbody (0.507)	other (0.579)		
		At1g43800	nucleus (0.760)	mitochondry (0.430)		
		At1g43800	nucleus (0.760)	mitochondry (0.430)		
		At1g52960	nucleus (0.960)	chloroplast (0.630)		
		At1g56070	mitochondry (0.360)	other (0.823)		
		At1g69290	chloroplast (0.840)	chloroplast (0.528)		
		At1g72560	cytoplasm (0.450)	other (0.872)		
		At2g17190	cytoplasm (0.650)	other (0.797)		
		At2g32920	endoplasmic reticulum (0.910) (C-terminal KDEL)	secretory pathway (0.920)	1-17 or 1-24	
		At2g36530	endoplasmic reticulum (0.600)	other (0.747)		
		At2g43710	microbody (0.495)	chloroplast (0.977)		
		At2g44350	mitochondry (0.883)	mitochondry (0.848)		
		At3g04600	nucleus (0.940	other (0.954)		
		At3g14440	chloroplast (0.905)	chloroplast (0.719)		
		At3g15730	microbody (0.634)	other (0.614)		
		At3g16857	nucleus (0.700)	other (0.376)		
		At3g57400	plasma membrane (0.830)	other (0.792)		
		At4g17260	cytoplasm (0.450)	chloroplast (0.703)		
		At4g31180	chloroplast (0.890)	chloroplast (0.876)		
		At4g36530	microbody (0.594)	other (0.828)		
		At4g37870	nucleus (0.880)	other (0.849)		
		At5g06450	microbody (0.640)	other (0.954)		
		At5g20080	mitochondry (0.859)	mitochondry (0.855)		
		At5g26710	endoplasmic reticulum (0.550)	chloroplast (0.552)		
		At5g28540	endoplasmic reticulum (0.910) (C-terminal HDEL)	secretory pathway (0.993)	1-27	
		At5g41550	chloroplast (0.520)	mitochondry (0.517)		

Colour code: proteins found at both steps 1 and 2 (orange)
proteins found at both steps 1 and 2 (blue)

(a) PSORT : http://psort.nibb.ac.jp/form.html [29]
(b) TargetP: http://www.cbs.dtu.dk/services/TargetP/ [30]
(c) Two sizes are indicated when different signal peptides are predicted by PSORT and TargetP. The first one is predicted with PSORT.
(d) Aramemnon: http://aramemnon.botanik.uni-koeln.de/ [31]
