# Supplementary material for: Evaluation of cell wall preparations for proteomics: a new procedure for purifying cell walls from Arabidopsis hypocotyls
Source: Plant Methods. 2006 May 27;2:10. doi: 10.1186/1746-4811-2-10 (PMC1524762; doi:10.1186/1746-4811-2-10)
Supplement: Additional data file 2 — Table 2 - Bioinformatic analysis of proteins extracted from cell walls of M. sativa stems [9] [file 1746-4811-2-10-S2.pdf]

Additional file 2: Bioinformatic analysis of proteins extracted from cell walls of M. sativa stems [9].

Data are from [9]. Proteins were extracted from *M. sativa* cell walls of stems as described in Figure 2. Two successive extractions were performed : step 1 using $CaCl_2$, and step 2 using LiCl. All proteins sequences were analyzed with bioinformatic softwares to predict their sub-cellular localization. When only partial protein sequences or no sequence are available, proteins are classified as "not predictable".

step 1: $CaCl_2$ extract

Predicted subcellular localization	Gene (*A. thaliana*)	Protein	PSORT (a)	TargetP (b)	Predicted signal peptide (c)	Aramemnon (d)
**transmembrane domain**		CAC34417	plasma membrane (0.460)	secretory pathway (0.986)	1-20	yes
		P16002	chloroplast (0.659), plasma membrane (0.650)	chloroplast (0.924)		yes
		P26291	plasma membrane (0.790), chloroplast (0.753)	chloroplast (0.797)		yes
**outside**	At1g09560	AAB51577	outside (0.609)	secretory pathway (0.960)	1-23	
	At3g62020	AAB51752	plasma membrane (0.685)	secretory pathway (0.970)	1-21	no
		BAA81904	plasma membrane (0.790)	secretory pathway (0.758)	1-39	no
		CAA09607	outside (0.820)	secretory pathway (0.996)	1-25 or 1-23	
		CAA10167	outside (0.790)	secretory pathway (0.963)	1-16 or 1-22	
		CAA10287	outside (0.585)	secretory pathway (0.867)	1-33	
		CAC44501	outside (0.820)	secretory pathway (0.985)	1-24	
		CAD29731	outside (0.714)	secretory pathway (0.982)	1-23 or 1-26	
	At1g49750	NP_175397	vacuole (0.750), outside (0.666)	secretory pathway (0.239)	1-15 or 1-22	
	At3g20820	NP_188718	vacuole (0.861), outside (0.820)	secretory pathway (0.752)	1-19	
	At4g34480	NP_195174	plasma membrane (0.460)	secretory pathway (0.942)	1-22	
	At5g14440	NP_196949	outside (0.786)	secretory pathway (0.941)	1-30	
	At5g51890	NP_200002	plasma membrane (0.685)	secretory pathway (0.991)	1-24	no
	At1g09750	NP_563851	outside (0.456)	secretory pathway (0.899)	1-21 or 1-23	
	At1g20850	NP_564126	vacuole (0.808), outside (0.786)	secretory pathway (0.987)	1-26	
	At4g33490	NP_567922	outside (0.820)	secretory pathway (0.913)	1-21	
		P36907	outside (0.757)	secretory pathway (0.985)	1-23	
		Q01806	plasma membrane (0.685)	secretory protein (0.983)	1-30	no
		Q9S8P4	outside (0.820)	secretory pathway (0.967)	1-20	
		S68805	outside (0.781)	secretory pathway (0.961)	1-24	
		T07086	outside (0.743)	secretory pathway (0.771)	1-19	
		T09642	vacuole (0.761), outside (0.657)	secretory pathway (0.981)	1-30 or 1-24	

**intracellular**		AAC49358	mitochondry (0.555)	chloroplast (0.628)
		AAG34872	microbody (0.540)	other (0.568)
		AAL77589	mitochondry (0.679)	chloroplast (0.765)
		CAA09177	microbody (0.640)	other (0.884)
	At1g16470	NP_173096	cytoplasm (0.450)	other (0.703)
	At1g23740	NP_173786	mitochondry (0.594)	chloroplast (0.568), mitochondry (0.442)
	At1g48590	NP_175292	mitochondry (0.480)	mitochondry (0.529)
	At1g67280	NP_176896	mitochondry (0.807	chloroplast (0.895)
	At2g15570	NP_179159	chloroplast (0.919)	chloroplast (0.979)
	At2g24940	NP_180066	cytoplasm (0.450)	other (0.890)
	At2g32520	NP_180811	microbody (0.570)	other (0.896)
	At3g20390	NP_188674	mitochondry (0.644)	chloroplast (0.497), mitochondry (0.371)
	At3g26060	NP_189235	mitochondry (0.679)	chloroplast (0.904)
	At3g43810	NP_189967	cytoplasm (0.650)	other (0.894)
	At3g55330	NP_191093	chloroplast (0.647)	chloroplast (0.966)
	At4g09010	NP_192640	chloroplast (0.512)	chloroplast (0.737)
	At5g01650	NP_195785	chloroplast (0.890)	other (0.719)
	At1g09310	NP_563841	cytoplasm (0.650)	other (0.906)
	At3g17440	NP_566578	endoplasmic reticulum (0.850)	other (0.736)
	At3g63190	NP_567141	mitochondry (0.838)	chloroplast (0.933)
	At5g20080	NP_568391	mitochondry (0.859)	mitochondry (0.855)
		O65194	chloroplast (0.876)	chloroplast (0.710)
		O65198	chloroplast (0.921)	chloroplast (0.941)
		P10933	endoplasmic reticulum (0.550)	chloroplast (0.907)
		P16048	mitochondry (0.870)	mitochondry (0.645)
		P16059	mitochondry (0.750)	chloroplast (0.307),
		P29450	chloroplast (0.950)	chloroplast (0.977)
		P49231	cytoplasm (0.450)	other (0.818)
		P51615	plasma membrane (0.615)	other (0.941)
		Q02610	cytoplasm (0.450)	other (0.404)
		Q42971	endoplasmic reticulum (0600)	other (0.608)
		Q43636	cytoplasm (0.450)	other (0.907)
			plasma membrane (0.650),	
		T06363	chloroplast (0.539)	chloroplast (0.505)
		T09286	mitochondry (0.920)	mitochondry (0.766)

**not predictable**	AAD34458	encoded by a truncated cDNA
	AAL06644	encoded by a truncated cDNA
	AAL15646	encoded by a truncated cDNA
	JC4780	data not available in usual databases
	JC4781	data not available in usual databases
	JC4782	data not available in usual databases
	S22489	encoded by a truncated cDNA
	T05957	encoded by a truncated cDNA
	T09165	encoded by a truncated cDNA

**step 2: LiCl extract**	**outside**	At1g49750	NP_175397	vacuole (0.850), outside (0.666)	secretory pathway (0.239)	1-15 or 1-22
			P36907	outside (0.757)	secretory pathway (0.985)	1-23
			T07086	outside (0.743)	secretory pathway (0.771)	1-19
			T07171	outside (0.820)	secretory pathway (0.981)	1-23
			T09665	outside (0.820)	secretory pathway (0.922)	1-28
	**intracellular**		AAD56659	mitochondry (0.483)	mitochondry (0.839)	
		At5g41550	NP_198970	chloroplast (0.520)	mitochondry (0.517)	
			T93508	endoplasmic reticulum (0.910) (C-term HDEL)	secretory pathway (0.991)	1-20
	**not predictable**		P04353	encoded by a truncated cDNA		

Colour code: proteins found at both steps 1 and 2

(a) PSORT : http://psort.nibb.ac.jp/form.html [29]
(b) TargetP: http://www.cbs.dtu.dk/services/TargetP/ [30]
(c) Two sizes are indicated when different signal peptides are predicted by PSORT and TargetP. The first one is predicted with PSORT.
(d) Aramemnon: http://aramemnon.botanik.uni-koeln.de/ [31]
