# Supplementary material for: Evaluation of cell wall preparations for proteomics: a new procedure for purifying cell walls from Arabidopsis hypocotyls
Source: Plant Methods. 2006 May 27;2:10. doi: 10.1186/1746-4811-2-10 (PMC1524762; doi:10.1186/1746-4811-2-10)
Supplement: Additional data file 3 — Table 3 – Bioinformatic analysis of proteins extracted from cell walls of C. albicans [20]. [file 1746-4811-2-10-S3.pdf]

Additional file 3: Bioinformatic analysis of proteins extracted from cell walls of *C. albicans* [20].

Data are from [20]. Proteins were extracted from C. albicans cell walls as described in Figure 3. Four successive extractions were performed : step 1 using SDS-DTT, step 2 using NaOH, step 3 using a β-1,3-glucanase and step 4 using an exochitinase. All proteins sequences were analyzed with bioinformatic programs to predict their sub-cellular localization. Proteins for which predictions by different bioinformatic programs are in conflict are classified as “not clear”.

	Predicted subcellular localization	Accession (a)	PSORT (b)	TargetP (c)	Predicted signal peptide (d)
**step 1: SDS-DTT extract**					
	**outside**	CA1541	outside (0.370)	secretory pathway (0.946)	1-18
		Phrp *			
	**intracellular**	CA0362	cytoplasm (0.650)	other (0.911)	
		CA0915	endoplasmic reticulum (0.910) (C-term HDEL)	secretory pathway (0.977)	1-29 or 1-32
		CA1015	mitochondry (0.541)	other (0.694), mitochondry (0.271)	
		CA1230	nucleus (0.760)	other (0.843)	
		CA1691	mitochondry (0.360)	other (0.789), mitochondry (0.227)	
		CA1755	endoplasmic reticulum (0.910) (C-term HDEL)	secretory pathway (0.955)	1-23
		CA2474	microbody (0.300)	other (0.798)	
		CA2857	nucleus (0.760)	other (0.842)	
		CA3081	nucleus (0.980)	other (0.885)	
		CA3208	cytoplasm (0.650)	mitochondry (0.663)	
		CA3483	mitochondry (0.360)	other (0.275), mitochondry (0.299)	
		CA3534	cytoplasm (0.450)	other (0.887)	
		CA3874	cytoplasm (0.450)	other (0.793)	
		CA4671	cytoplasm (0.450)	other (0.834)	
		CA4765	mitochondry (0.853)	mitochondry (0.716)	
		CA4844	endoplasmic reticulum (0.550)	other (0.704)	
		CA4862	mitochondry (0.360)	other (0.934)	
		CA4959	nucleus (0.600)	other (0.923)	
		CA5135	cytoplasm (0.450)	other (0.928)	
		CA5180	cytoplasm (0.450)	other (0.797)	
		CA5892	mitochondry (0.471)	mitochondry (0.637)	
		CA5950	microbody (0.800)	other (0.604)	
	**not predictable**	CA2810	not present in the database		
**step 2: NaOH extract**	**outside**	Hsp150p/Pir2p*			
	**intracellular**	CA1691	mitochondry (0.360)	other (0.789), mitochondry (0.227)	
		CA2474	microbody (0.300)	other (0.798)	
		CA3483	mitochondry (0.360)	other (0.275), mitochondry (0.299)	
		CA3874	cytoplasm (0.450)	other (0.793)	
		CA4765	mitochondry (0.853)	mitochondry (0.716)	
		CA5180	cytoplasm (0.450)	other (0.797)	
		CA5892	mitochondry (0.471)	mitochondry (0.637)	

**step 3: β-1,3-glucanase extract**	**outside**	putative β-1,3-glucanase*		
		Hsp150p/Pir2p*		
		Phrp *		
	**intracellular**	CA1691	mitochondry (0.360)	other (0.789), mitochondry (0.227)
		CA3874	cytoplasm (0.450)	other (0.793)
		CA5892	mitochondry (0.471)	mitochondry (0.637)
**step 4: exochitinase extract**	**outside**	Hsp150p/Pir2p*		
		Phrp *		
	**intracellular**	CA1691	mitochondry (0.360)	other (0.789), mitochondry (0.227)
		CA2474	microbody (0.300)	other (0.798)
		CA3874	cytoplasm (0.450)	other (0.793)
		CA5180	cytoplasm (0.450)	other (0.797)
		CA5892	mitochondry (0.471)	mitochondry (0.637)

colour code:
- (orange) proteins found in at least 2 fractions
- (blue) proteins found in at least 2 fractions

* proteins that failed to be identified to already annotated *C. albicans* proteins
(a) http://genolist.pasteur.fr/CandidaDB/
(b) PSORT : http://psort.nibb.ac.jp/form.html [29]
(b) TargetP: http://www.cbs.dtu.dk/services/TargetP/ [30]
(d) Two sizes are indicated when different signal peptides are predicted by PSORT and TargetP. The first one is predicted with PSORT.
