# Supplementary material for: Evaluation of cell wall preparations for proteomics: a new procedure for purifying cell walls from Arabidopsis hypocotyls
Source: Plant Methods. 2006 May 27;2:10. doi: 10.1186/1746-4811-2-10 (PMC1524762; doi:10.1186/1746-4811-2-10)
Supplement: Additional data file 4 — Table 4 - Bioinformatic analysis of proteins extracted from cell walls of A. thaliana etiolated hypocotyls with salts. [file 1746-4811-2-10-S4.pdf]

Additional file 4: Bioinformatic analysis of proteins extracted from cell walls of *A. thaliana* etiolated hypocotyls with salts.

Proteins were extracted from A. thaliana cell walls of etiolated hypocotyls as described in Figure 5. Two successive extractions were performed : step 1 using $CaCl_2$, and step 2 using LiCl. All proteins sequences were analyzed with bioinformatic programs to predict their sub-cellular localization. Proteins for which predictions by different bioinformatic programs are in conflict are classified as “not clear”.

step 1: CaCl2 extract	Predicted subcellular localization	Gene (*A. thaliana*)	PSORT (a)	TargetP (b)	Predicted signal peptide (c)	Aramemnon (d)
	**transmembrane domain**	At1g23480	plasma membrane (0.919)	secretory pathway (0.875)	1-29	yes
		At1g54010	plasma membrane (0.685)	secretory pathway (0.991)	1-29	yes
		At1g55850	plasma membrane (0.600)	other (0.787)		yes
		At1g62660	plasma membrane (0.790)	other (0.822)		yes
		At1g79680	outside (0.820)	secretory pathway (0.929)	1-26 or 1-24	yes
		At3g20580	plasma membrane (0.600)	secretory pathway (0.860)	1-35	yes (GPI anchor)
		At3g45970	endoplasmic reticulum (0.820)	secretory pathway (0.854)	1-20	yes
		At4g15320	plasma membrane (0.600)	other (0.879)		yes
	**outside**	At1g02335	plasma membrane (0.685)	secretory pathway (0.933)	1-22	no
		At1g03220	outside (0.820)	secretory pathway (0.912)	1-22	
		At1g03230	outside (0.820)	secretory pathway (0.910)	1-23	
		At1g09560	outside (0.609)	secretory pathway (0.960)	1-23	
		At1g09750	outside (0.456)	secretory pathway (0.899)	1-21 or 1-23	
		At1g11580	outside (0.820)	secretory pathway (0.423)	1-34	
		At1g18970	mitochondry (0.850), plasma membrane (0.650)	secretory pathway (0.932)	1-27	no
		At1g20190	outside (0.820)	secretory pathway (0.961)	1-20	
		At1g28290	outside (0.681)	secretory pathway (0.483)	1-24	
		At1g29670	outside (0.820)	secretory pathway (0.987)	1-24	
		At1g33590	outside (0.738)	secretory pathway (0.963)	1-24	no
		At1g65590	outside (0.819)	secretory pathway (0.948)	1-24	
		At1g68560	outside (0.820)	secretory pathway (0.982)	1-27	
		At1g73260	outside (0.542)	secretory pathway (0.985)	1-26	
		At1g76160	outside (0.820)	secretory pathway (0.984)	1-23	
		At1g78830	outside (0.820)	secretory pathway (0.962)	1-22	
		At1g78850	outside (0.820)	secretory pathway (0.973)	1-22	
		At1g78860	outside (0.652)	secretory pathway (0.981)	1-22	
		At2g05580	outside (0.820)	secretory pathway (0.993)	1-20	no
		At2g18140	outside (0.820)	secretory pathway (0.695)	1-20 or 1-16	
		At2g28790	outside (0.705)	secretory pathway (0.990)	1-24 or 1-21	
		At2g30210	outside (0.820)	secretory pathway (0.983)	1-25	
		At2g38530	plasma membrane (0.685)	secretory pathway (0.967)	1-23	no
		At3g08030	outside (0.820)	secretory pathway (0.985)	1-21	
		At3g13790	outside (0.494)	secretory pathway (0.969)	1-20 or 1-28	
		At3g14220	outside (0.609)	secretory pathway (0.926)	1-20 or 1-28	
		At3g14310	plasma membrane (0.460)	secretory pathway (0.227)	1-40 or 1-37	no
		At3g16850	outside (0.820)	secretory pathway (0.965)	1-19 or 1-20	
		At3g18080	outside (0.370)	secretory pathway (0.374)	1-23	

	At3g20370	outside (0.633)	secretory pathway (0.965)	1-25	
	At3g20820	outside (0.820)	secretory pathway (0.752)	1-19	
	At3g21770	outside (0.820)	secretory pathway (0.967)	1-27	
	At3g22640	outside (0.820)	secretory pathway (0.996)	1-22	
	At3g24480	outside (0.795)	secretory pathway (0.978)	1-25	
	At3g25700	endoplasmic reticulum (0.820)	secretory pathway (0.425)	1-23	
	At3g43270	outside (0.820)	secretory pathway (0.979)	1-24	
	At3g49120	outside (0.609)	secretory pathway (0.604)	1-28 or 1-29	
	At3g54400	outside (0.595)	secretory pathway (0.409)	1-19	
	At3g55260	outside (0.738)	secretory pathway (0.805)	1-22 or 1-20	
	At4g08950	outside (0.820)	secretory pathway (0.871)	1-21	
	At4g12880	outside (0.820)	secretory pathway (0.952)	1-18 or 1-26	
	At4g13340	outside (0.820)	secretory pathway (0.945)	1-20	
	At4g16500	outside (0.820)	secretory pathway (0.929)	1-22	
	At4g18970	plasma membrane (0.685)	secretory pathway (0.980)	1-22	no
	At4g29270	outside (0.820)	secretory pathway (0.983)	1-26	
	At4g30170	outside (0.695)	secretory pathway (0.980)	1-25	
	At4g33220	outside (0.709)	secretory pathway (0.919)	1-19	
	At5g02260	plasma membrane (0.685)	secretory pathway (0.969)	1-20	no
	At5g06230	plasma membrane (0.790)	secretory pathway (0.744)	1-38	
	At5g06860	outside (0.733)	secretory pathway (0.964)	1-21	
	At5g07030	outside (0.820)	secretory pathway (0.559)	1-19	
	At5g09440	outside (0.820)	secretory pathway (0.812)	1-19 or 1-23	
	At5g10770	plasma membrane (0.760)	secretory pathway (0.276)	1-25	
	At5g11420	outside (0.820)	secretory pathway (0.989)	1-22	
	At5g12940	outside (0.820)	secretory pathway (0.985)	1-29 or 1-26	
	At5g23210	outside (0.820)	secretory pathway (0.992)	1-25	
	At5g25460	outside (0.820)	secretory pathway (0.979)	1-19	
	At5g26260	outside (0.547)	secretory pathway (0.926)	1-22 or 1-23	
	At5g26280	outside (0.528)	secretory pathway (0.963)	1-22 or 1-23	
	At5g34850	outside (0.748)	secretory pathway (0.870)	1-16 or 1-22	
	At5g34940	outside (0.820)	secretory pathway (0.996)	1-21	
	At5g43060	outside (0.685)	secretory pathway (0.991)	1-21	
	At5g44380	plasma membrane (0.811)	secretory pathway (0.439)	1-32	no
	At5g63810	outside (0.819)	secretory pathway (0.887)	1-29	
	At5g64260	outside (0.685)	secretory pathway (0.966)	1-19 or 1-23	
**intracellular**	At1g67090*	chloroplast (0.923)	chloroplast (0.769)		
	At3g09260	endoplasmic reticulum (0.910) (C-term KDEL)	secretory pathway (0.952)	1-24	
	At3g16880	cytoplasm (0.450)	other (0.625)		
	At4g15440	microbody (0.540)	other (0.891)		
	At4g22165	microbody (0.640)	other (0.493)		
	At4g23670	microbody (0.560)	other (0.847)		
	At4g28520°	outside (0.820)	secretory pathway (0.962)	1-23	
	At5g38410*	outside (0.370)	chloroplast (0.741)		
	At5g38420*	endoplasmic reticulum (0.550)	chloroplast (0.806)		
	At5g38430*	outside (0.370)	chloroplast (0.807)		
	At5g44120°	outside (0.700)	secretory pathway (0.975)	1-24 or 1-23	
**not clear**	At2g21540	plasma membrane (0.700)	other (0.945)		no
	At3g26380	outside (0.690)	other (0.355), secretory pathway (0.168)	1-32	yes
	At3g32980	mitochondry (0.660)	secretory pathway (0.356)	1-29	

**step 2: LiCl extract**	**transmembrane domain**	At1g54010	plasma membrane (0.685)	secretory pathway (0.991)	1-29	yes
		At1g73620	Golgi (0.900), plasma membrane (0.790)	other (0.532)		yes
		At4g16590	plasma membrane (0.600)	other (0.879)		yes
		At5g20630	plasma membrane (0.460)	secretory pathway (0.922)	1-20	yes
	**outside**	At1g03220	outside (0.820)	secretory pathway (0.912)	1-22	
		At1g03230	outside (0.820)	secretory pathway (0.910)	1-23	
		At1g09750	outside (0.456)	secretory pathway (0.899)	1-21 or 1-23	
		At1g17860	outside (0.690)	secretory pathway (0.910)	1-19	
		At1g29670	outside (0.820)	secretory pathway (0.987)	1-24	
		At1g47128	outside (0.771)	secretory pathway (0.993)	1-21	
		At1g68560	outside (0.820)	secretory pathway (0.982)	1-27	
		At1g73260	outside (0.542)	secretory pathway (0.985)	1-26	
		At1g78850	outside (0.820)	secretory pathway (0.973)	1-22	
		At2g05580	outside (0.820)	secretory pathway (0.993)	1-20	
		At2g22170	outside (0.820)	secretory pathway (0.990)	1-21	
		At2g28790	outside (0.705)	secretory pathway (0.994)	1-24 or 1-21	
		At2g34700	outside (0.432)	secretory pathway (0.790)	1-23 or 1-28	
		At3g14310	plasma membrane (0.460)	secretory pathway (0.227)	1-40 or 1-37	no
		At3g16850	outside (0.820)	secretory pathway (0.965)	1-19 or 1-20	
		At3g21770	outside (0.820)	secretory pathway (0.967)	1-27	
		At3g49120	outside (0.609)	secretory pathway (0.604)	1-28 or 1-29	
		At3g54400	outside (0.595)	secretory pathway (0.409)	1-19	
		At4g30170	outside (0.695)	secretory pathway (0.980)	1-25	
		At4g34980	outside (0.820)	secretory pathway (0.980)	1-20	
		At5g06860	outside (0.733)	secretory pathway (0.964)	1-21	
		At5g07030	outside (0.820)	secretory pathway (0.559)	1-19	
		At5g09440	outside (0.820)	secretory pathway (0.812)	1-19 or 1-23	
		At5g11420	outside (0.820)	secretory pathway (0.989)	1-22	
		At5g25460	outside (0.820)	secretory pathway (0.979)	1-19	
		At5g26280	outside (0.528)	secretory pathway (0.963)	1-22 or 1-23	
		At5g51260	outside (0.820)	secretory pathway (0.938)	1-19	
		At5g59090	outside (0.820)	secretory pathway (0.978)	1-24	
		At5g66390	outside (0.820)	secretory pathway (0.973)	1-23	
	**intracellular**	At5g20830	microbody (0.475)	other (0.669)	1-20	
	**not clear**	At3g42160	plasma membrane (0.650)	other (0.925)		no

colour code:

proteins found at both steps 1 and 2

proteins found at both steps 1 and 2

* small subunit of RUBISCO

° homolog to storage proteins, most probably vacuolar localization

(a) PSORT : http://psort.nibb.ac.jp/form.html [29]

(b) TargetP: http://www.cbs.dtu.dk/services/TargetP/ [30]

(c) Two sizes are indicated when different signal peptides are predicted by PSORT and TargetP. The first one is predicted with PSORT.

(d) Aramemnon: http://aramemnon.botanik.uni-koeln.de/ [31]
