# Supplementary material for: Evaluation of cell wall preparations for proteomics: a new procedure for purifying cell walls from Arabidopsis hypocotyls
Source: Plant Methods. 2006 May 27;2:10. doi: 10.1186/1746-4811-2-10 (PMC1524762; doi:10.1186/1746-4811-2-10)
Supplement: Additional data file 5 — Table 5 - Bioinformatic analysis of proteins extracted from cell walls of A. thaliana etiolated hypocotyls with SDS and DTT. [file 1746-4811-2-10-S5.pdf]

Additional file 5: Bioinformatic analysis of proteins extracted from cell walls of *A. thaliana* etiolated hypocotyls with SDS and DTT.

Proteins were extracted from *A. thaliana* cell walls of etiolated hypocotyls as described in Figure 6. One extraction was performedusing SDS and DTT. All proteins sequences were analyzed with bioinformatic programs to predict their sub-cellular localization. Proteins for which predictions by different bioinformatic programs are in conflict are classified as “not clear”.

step 3: SDS-DTT

Predicted subcellular localization	Gene (*A. thaliana*)	PSORT (a)	TargetP (b)	Predicted signal peptide (c)	Aramemnon (d)
**transmembrane domain**	At2g25300	chloroplast thylakoid membrane (0.765), plasma membrane (0.650)	chloroplast (0.573)		yes
	At4g33230	chloroplast thylakoid membrane (0.615), plasma membrane (0.600)	other (0.932)		yes
	At4g25080	endoplasmic reticulum (0.600), mitochondry (0.453), chloroplast (0.376)	chloroplast (0.866)		yes
	At2g24820	mitochondry (0.791), microbody (0.635), chloroplast (0.500)	chloroplast (0.897)		yes
	At4g15820	mitochondry (0.861), chloroplast (0.660), plasma membrane (0.650)	mitochondry (0.537)		yes
	At1g72990	outside (0.820)	mitochondry (0.957)	1-29	yes
	At1g27200	plasma membrane (0.790)	other (0.521)		yes
	At3g05610	plasma membrane (0.790)	mitochondry (0.537)		yes
	At3g61270	plasma membrane (0.790)	other (0.843)		yes
	At4g15320	plasma membrane (0.600)	other (0.879)		yes
	At4g16590	plasma membrane (0.600)	other (0.879)		yes
	At5g41390	plasma membrane (0.600)	mitochondry (0.750), other (0.544)		yes
**outside**	At1g66270	outside (0.820)	secretory pathway (0.976)	1-24	
	At1g71695	outside (0.820)	secretory pathway (0.829)	1-22 or 1-31	
	At2g46570	outside (0.370)	secretory pathway (0.868)	1-20 or 1-29	
	At3g14040	outside (0.820)	secretory pathway (0.984)	1-23 or 1-25	
	At4g11310	outside (0.820)	secretory pathway (0.995)	1-18 or 1-23	
	At4g12880	outside (0.820)	secretory pathway (0.952)	1-18 or 1-26	
	At4g28790	outside (0.820)	secretory pathway (0.726)	1-17 or 1-24	
	At4g33810	outside (0.820)	secretory pathway (0.910)	1-24	
	At5g07030	outside (0.820)	secretory pathway (0.559)	1-19	
	At5g25460	outside (0.820)	secretory pathway (0.979)	1-19	
	At5g44020	mitochondry (0.850), outside (0.820)	secretory pathway (0.845)	1-16 or 1-21	

**intracellular**	ArthCp029	chloroplast			
	ArthCp030	chloroplast			
	At1g55490	chloroplast (0.864)	chloroplast (0.979)		
	At1g67090*	chloroplast (0.923)	chloroplast (0.769)		
	At2g21170	chloroplast (0.888)	chloroplast (0.950)		
	At3g04350	chloroplast (0.842)	other (0.635), chloroplast (0.451)		
	At3g13470	chloroplast (0.950)	chloroplast (0.924)		
	At3g53460	chloroplast (0.903)	chloroplast (0.903)		
	At4g15040	chloroplast (0.496)	other (0.625), chloroplast (0.289)		
	At4g20360	chloroplast (0.950)	chloroplast (0.975)		
	At5g20720	chloroplast (0.895)	chloroplast (0.901)		
	At5g54190	chloroplast (0.924)	chloroplast (0.957)		
	At3g09260	endoplasmic reticulum (0.910) (C-term KDEL)	secretory pathway (0.952)	1-24	
	At4g28520°	outside (0.820)	secretory pathway (0.962)	1-23	
	At5g44120°	outside (0.700)	secretory pathway (0.975)	1-24 or 1-23	
	At1g49240	cytoplasm (0.450)	other (0.891)		
	At3g18780	cytoplasm (0.450)	other (0.883)		
	At4g11850	cytoplasm (0.450)	other (0.806)		
	At5g17920	cytoplasm (0.450)	other (0.713)		
	At4g02520	microbody (0.520)	other (0.556)		
	At2g13890	endoplasmic reticulum (0.850)	other (0.609)		
	At1g13440	microbody (0.539)	other (0.610)		
	At1g60120	microbody (0.704)	other (0.753)		
	At1g78380	microbody (0.640)	other (0.523)		
	At3g02230	microbody (0.603)	other (0.765)		
	At3g04120	microbody (0.472)	other (0.619)		
	At3g60120	microbody (0.640)	other (0.712)		
	At5g58690	microbody (0.499)	other (0.598)		

**not clear**	At3g49400	plasma membrane (0.650)	other (0.338)		no
	At5g37470	plasma membrane (0.600)	other (0.973)		no

colour code:

proteins also extracted by $CaCl_2$ or LiCl

proteins also extracted by $CaCl_2$ or LiCl

proteins also extracted by $CaCl_2$ or LiCl

* small subunit of RUBISCO
° homolog to storage proteins, most probably vacuolar localization
(a) PSORT : http://psort.nibb.ac.jp/form.html [29]
(b) TargetP: http://www.cbs.dtu.dk/services/TargetP/ [30]
(c) Two sizes are indicated when different signal peptides are predicted by PSORT and TargetP. The first one is predicted with PSORT.
(d) Aramemnon: http://aramemnon.botanik.uni-koeln.de/ [31]
